# Microbial, phytochemical, toxicity analyses and antibacterial activity against multidrug resistant bacteria of some traditional remedies sold in Buea Southwest Cameroon

**DOI:** 10.1186/s12906-019-2563-z

**Published:** 2019-06-26

**Authors:** Moses Njutain Ngemenya, Ghogo Gail Rinda Djeukem, Kennedy Dohjinga Nyongbela, Petuel Ndip Ndip Bate, Smith Borakaeyabe Babiaka, Elvis Monya, Rudolf Khundou Kanso

**Affiliations:** 1 Department of Biochemistry and Molecular Biology, Faculty of Science, University of Buea, P.O. Box 63, Buea, South West Region Cameroon; 2 Department of Chemistry, Faculty of Science, University of Buea, P.O. Box 63, Buea, South West Region Cameroon; 3 Biotechnology Unit, Faculty of Science, University of Buea, P.O. Box 63, Buea, South West Region Cameroon

**Keywords:** Resistance, Antibacterial, Traditional medicine remedy, Toxicity, Phytochemistry

## Abstract

**Background:**

Traditional medicine remedies are commonly used for treatment of diverse ailments including bacterial infections. The activity against resistant bacteria and safety of some remedies sold as anti-infective treatments in market places in Buea, Southwest Cameroon were investigated as potential alternative treatment to counter increasing antibiotic resistance.

**Methods:**

Ten remedies were purchased, their components documented and microbial load estimated. Methanol extracts of the remedies were tested for antibacterial activity by disc diffusion and microdilution. Cytotoxicity was evaluated on monkey kidney epithelial cells (LLC-MK2) while acute oral toxicity was done in BALB/c mice for the bactericidal extract. Extracts were further analysed using phytochemical tests.

**Results:**

All the remedies had microbial loads above the acceptable limit of 10^5^ CFU/g. The highest activity was produced by extracts of four remedies (TP 1, 2, 4, 6a, 6b) against all clinical isolates among which three were active against four control strains. Zones of inhibition ranged from 8 to 27 mm. Two of the four extracts produced zones ≥20 mm against multidrug resistant clinical isolates of *Citrobacter freundii* and *Escherichia coli* but were less active compared to Gentamycin positive control (*P* < 0.0001–0.0014). The most active extracts also recorded minimum inhibitory concentrations of 1 to 4 mg/mL. One of them (TP2) was bactericidal against a clinical isolate of methicillin–resistant *Staphylococcus aureus* with a minimum bactericidal concentration of 8 mg/mL. Extracts of six remedies did not show cytotoxicity and no mortality or adverse effect was recorded in the acute oral toxicity test. Phytochemical screening showed the most active extracts contained relatively high amounts of alkaloids and flavonoids.

**Conclusion:**

Only four of the eight remedies tested showed activity against multidrug resistant bacteria suggesting some of these remedies may not be effective against bacterial infections. Production and handling methods should be improved and the product quality controlled to ensure biosecurity. The remedies which were both active and non-toxic should be further investigated including in vivo experiments to assess their efficacy.

## Background

Pathogenic microorganisms (bacteria, viruses, parasites and fungi) contribute significantly to the global disease burden. In particular, bacterial infections account for high morbidity and mortality with respiratory and diarrhoeal diseases (caused by bacteria), and tuberculosis featuring among the leading causes of death. Furthermore, communicable diseases account for more than 50% of annual mortality in the lower income countries like Cameroon [1]. Presently resistance to antibiotics is threatening chemotherapy of bacterial infections. It has led to the emergence of extensively drug-resistant and pandrug-resistant phenotypes in a good number of bacterial species and has been documented in virtually all antibiotic classes [2, 3]. This situation has been compounded by the slow pace of development of new antibiotics whereby no new classes have been introduced into clinical use in the last two decades [4] except for the recently discovered teixobactin which is undergoing development [5]. The infectious disease burden is highest in developing countries particularly in Sub-Saharan Africa, necessitating high antibiotic use which in turn leads to high levels of resistance [6]. Overcoming the antibiotic resistance onslaught requires discovery and development of new efficacious antibiotics and alternative treatments based on natural products among other approaches.

Traditional medicines or remedies are widely used as alternative or complementary treatment to pharmaceutical products and the global market is growing rapidly [7]. These remedies serve as a primary source of healthcare for treatment of many ailments including infectious diseases. However, some of these remedies have many limitations which include unascertained quality, safety, efficacy, lack of regulation among others [7].Various remedies are routinely sold in public places and local markets in Cameroon and are widely consumed as in most developing countries [8]. Another limitation is a gross lack of information on their composition due to no independent scientific data on their production. A few studies on such remedies have shown a mixed picture. One study of herbal products reported considerable antibacterial activity indicating potential therapeutic benefit [9], whereas another found weak or no antibacterial activity alongside significant bacterial contamination [10]. Furthermore, a study reported no antibacterial activity in four products [11]. Toxicity has been reported for a good number of herbal products particularly hepatoxicity; this is possible because this is the first major organ exposed to these products consumed principally by the oral route at unregulated doses [12].

Most studies on traditional remedies have been limited to in vitro experiments; in vivo efficacy studies are rare despite findings of significant activity in vitro [9, 13]. Significant antiviral activity was reported in vitro and in vivo in mice for a crude extract of a complex herbal preparation formulated from three antiviral plants [14]. Commonly used commercial antimalarial herbal preparations in Ghana were found to show chemosuppressive activity in vivo in *Plasmodium berghei* infected mice [15]. An approved study involving humans recorded good antibacterial activity in vitro for *Plantago lanceolata* herbal tea used in European countries for the local treatment of oral or pharyngeal irritations; and also significant antibacterial activity in vivo in human subjects when used as a mouth rinse [16]. These findings justify in vivo efficacy studies of traditional medicine preparations which have shown significant activity in vitro to further assess their therapeutic potential.

This study therefore embarked on investigating the microbial content, phytochemical composition, antibacterial activity and toxicity of selected anti-infective traditional medicine remedies sold in local markets in Buea municipality, Southwest Cameroon. This was aimed at evaluating the suitability of these products as alternative antibacterials; and to identify remedies which could be further developed into improved traditional medicine therapeutics.

## Methods

### Study design

This was a laboratory-based experimental study. A selection of traditional medicine remedies were purchased based on information provided by the sellers on the infectious diseases treated. The bacterial loads of the preparations were determined to ascertain their microbial safety. Clinical isolates and control strains of bacteria were characterised for antibiotic susceptibility to identify resistant strains. Methanol crude extracts of the powders were prepared, phytochemical constituents determined and together with the liquid preparations (not extracted) were screened for activity against the characterised bacteria. Toxicity of active extracts was investigated on a cell line and in vivo in mice. Experimental data were analysed by comparison with reference data.

### Collection of traditional remedies

We approached sellers of traditional medicine remedies in the local markets (Muea and Central markets) in Buea municipality, Southwest Cameroon, who consented to provide information on their remedies displayed for sale. Information on the name, source, disease(s) treated, component(s) of the remedy and their local name(s) were noted. Ten anti-infective remedies were purchased. Where taxonomic nomenclature of component(s) was provided, this was crosschecked in online plant databases and publications on ethnobotanical surveys in the area of origin of the remedy. The seller or respondent was requested to provide specimens (leaves, seeds, and flower of plants) for identification. Preliminary identification of the samples was done by botanists, Professor Chuyong George and Dr. Neba Godlove in the Department of Plant and Animal Physiology, University of Buea, Cameroon. The identities were confirmed by Mr. Ndive Elias using voucher specimens in the Biodiversity and Conservation Centre in Limbe and the National Herbarium of Cameroon in Yaounde and the voucher specimen numbers noted. Where the taxonomic names of the components could not be established such remedies were excluded from the study; but their identification will be pursued further to the source of the product subsequently.

### Determination of microbial content of remedies

Presence of bacterial contaminants in the remedies was determined as described [10]. Five dilutions of 100, 50, 25, 10 and 1% (v/v) of the liquid remedy were made. For the powders, 10 mg of each was put in 1 mL of 0.85% saline, vortexed and allowed to stand. The supernatant was collected and diluted same as liquid remedies. Then 100 μL of each dilution was uniformly spread on nutrient agar and incubated (DHP-9052, England) at 37 °C for 24 h. The number of colony forming units (CFUs) were counted and the microbial load calculated in CFUs/g or CFUs/mL. Gram staining was done for microscopic analysis as described [17] using a loop of cells and visualized under oil immersion.

### Preparation of extracts and phytochemical analysis

Extracts of the remedies were prepared as described [18]. Briefly, weighed amounts (14 to 80 g) of the powders were macerated three times in methanol for 72 h with intermittent stirring, filtered and the filtrate concentrated by rotary evaporation (Buchi Rotavapor R200, Switzerland). The extracts were dried at room temperature and stored at 4 °C until used. Phytochemical analysis was performed for each extract to determine chemical classes of secondary metabolites present in them and their relative amounts based on qualitative observations produced by specific chemical tests for alkaloids, flavonoids, steroids, tannins and triterpenoids [19].

### Source and characterisation of bacteria

Ten resistant strains of nine pathogenic bacteria species (*Citrobacter freundii*, *Citrobacter youngae*, *Enterobacter cloacae*, *Escherichia coli*, *Proteus mirabilis*, *Proteus vulgaris*, *Providencia rettgeri*, *Salmonella typhi*, *Salmonella* sp. and Methycillin-resistant *Staphylococcus aureus* (MRSA) were obtained from clinical specimens in Buea Regional Hospital annex, a regional referral health facility in the Southwest region of Cameroon. The cells were characterized by bacteriologists of the hospital by microscopy, susceptibility to reference antibiotics, appropriate culture methods and biochemical tests using the API 20E kit (Biomerieux SA, France). Pure stocks of cells were then stored in 10% glycerol in Mueller Hinton broth (Liofilchem, Italy) at − 20 °C during the study period. The following control strains were obtained from BEI Resources and American Type Culture Collection (USA): *Escherichia coli* (NR 32771), *P. aeruginosa* (ATCC 27853), *Staphylococcus aureus* (NR-46003), *Staphylococcus epidermidis* (NR-46376), *Salmonella enterica* (NR-515) and *Salmonella enterica* (NR-4311).

For the purpose of this study, the clinical isolates were re-tested for antibiotic susceptibility to determine the magnitude of their resistance, together with the control strains. For experimental use, working near the freezer the stored cells were retrieved, the frozen stock rapidly scraped using an autoclaved toothpick and deposited on the surface of nutrient agar; and the stock returned immediately into the freezer. A wire loop was flamed and then used to streak on nutrient agar. Susceptibility was done on the clinical isolates as described by the Clinical and Laboratory Standard Institute [20], using discs of 12 standard antibiotics (Liofilchem, Italy), from eight chemical classes selected for this study: Amikacin (30 μg), Ceftriaxone (30 μg), Cefuroxime (30 μg), Cefotaxime (30 μg), Ciprofloxacin (5 μg), Chloramphenicol (30 μg), Gentamicin (10 μg), Imipenem (10 μg), Nitrofurantoin (300 μg), Norfloxacin (10 μg), Tetracycline (30 μg), Trimethoprim (5 μg).

### Determination of antibacterial activity of extracts

All experiments were performed under sterile conditions using sterilized glassware and materials. Freshly sub-cultured bacterial cells were used in all experiments. The disc diffusion test was performed as described [18] with some modifications. Stock solutions of the extracts (10 mg/100 μL dimethylsufoxide, DMSO) were prepared while the liquid remedies were tested undiluted. Bacterial suspension (0.5 McFarland) was spread on Mueller Hinton agar (Liofilchem, Italy) and allowed for 3–5 min to dry. Six millimetre sterile Whatman filter paper discs (number 9) were gently fixed at labelled positions on the agar surface. Then 10 μL of test solution (1 mg extract) was transferred onto each disc. Positive (appropriate standard antibiotic) and negative (10 μL DMSO) controls were included, the plates kept for 30 min at room temperature, incubated as above and zones of inhibition measured.

The minimum inhibitory concentrations (MIC) of extracts were determined using the microdilution method as described [18] with one modification: experimental changes in microtitre plate wells were recorded by spectrophotometry. A stock solution of each extract was prepared (40 mg dissolved in 200 μL DMSO and 800 μL Mueller Hinton broth added); then diluted to obtain test solutions from 40 to 1 mg/mL. The assay was set up in a 96 well microtitre plate in duplicate wells as follows: 50 μL test solution, 130 μL Mueller Hinton broth followed by 20 μL bacterial cell suspension (6 × 10^5^ CFU/mL final density). Positive (20 μg/mL Gentamicin) and negative (0.85% saline) controls were included. Final test concentrations were 10, 8, 6, 4, 2, 1, 0.5 and 0.25 mg/mL respectively containing 5% DMSO. The optical densities (ODs) of wells were read, the plate incubated as above and the OD read again at 595 nm (Emax-Molecular Devices Corporation, USA).

The MIC was calculated from % growth of bacteria relative to the negative control using the formula below:

$$ \%\mathrm{Growth}=\frac{\ \mathrm{Change}\ \mathrm{in}\ \mathrm{OD}\ \mathrm{of}\ \mathrm{Test}\ \mathrm{Substance}\ }{\mathrm{Change}\ \mathrm{in}\ \mathrm{OD}\ \mathrm{of}\ \mathrm{Negative}\ \mathrm{Control}}\times 100. $$

where change in OD = OD at 24 h – OD at 0 h. MIC is taken as the lowest concentration at which % growth becomes approximately constant with no further decrease in growth. To determine minimum bactericidal concentration (MBC), a 1:10 dilution in fresh broth of MIC well contents were re-incubated and percentage growth calculated from ODs before and after incubation. The lowest concentration without bacterial growth was recorded as MBC using the formulas: change in OD = OD at 24 h – OD at 0 h and

$$ \%\mathrm{Growth}\ \mathrm{of}\ \mathrm{bacteria}\ \mathrm{cells}=\frac{\mathrm{OD}\ \mathrm{at}\ 24\mathrm{hr}-\mathrm{OD}\ \mathrm{at}\ 0\mathrm{hr}}{\mathrm{OD}\ \mathrm{at}\ 0\mathrm{hr}}\times 100 $$

### Cytotoxicity test

The cytotoxicity of the extracts was investigated as described [21] using monkey kidney epithelial cells, LLCMK2 Original (ATCC® CCL7™), (Virginia, USA). All reagents were from Sigma Aldrich (USA). The cells were cultured to confluence in complete RPMI-1640 medium, CCM (containing NaHCO_3_, supplemented with 25 Mm HEPES, 0.3 g γ-irradiated L-glutamine powder, 10% heat inactivated new born calf serum, 200 units/mL penicillin and 200 μg/mL streptomycin and 0.25 μg/mL amphotercin B, pH 7.4) in a T-shaped flask in a 5% CO_2_ incubator in humidified air at 37 °C (Heracell 150i, USA). The medium was decanted, washed off twice with incomplete (calf serum-free) RPMI-1640 medium (ICM), and cells dislodged with × 5 trypsin-EDTA. The cells were then centrifuged at 125 x g for 10 min (Eppendorf 5810R, Germany). The supernatant was discarded; the cells re-suspended in fresh ICM and quantified using a haemocytometer (Hausser Scientific, USA) and an inverted microscope (Nikon Eclipse TS100, China). The cells were diluted to 30,000/mL with CCM, 100 μL seeded in a 96-well flat bottom microtitre plate in duplicate and incubated as above for 3 days for cells to grow and become fully confluent.

Stock solutions of extracts (25 mg/mL DMSO) were prepared and diluted with CCM to 2 mg/mL in duplicate microtitre wells. The seeded cells were checked for confluence by microscopy. Then 100 μL of extract was transferred into respective wells giving final concentration of 1 mg/mL. Positive (30 μM auranofin) and negative (2% DMSO in CCM) controls were included. The plates were incubated as above for 5 days with daily microscopic examination for toxicity. Extracts that were cytotoxic were serially diluted in CCM and re-incubated same as above with freshly cultured cells at final extract concentrations of 15–1000 μg/mL. Thereafter the medium was discarded and wells decolourised by shaking with ICM (IKA Labortechnik KS125 basic shaker) at 600 rpm for 5 min three times. Then 100 μL of 5 mg/mL MTT in ICM was added, the plate incubated as above for 30 min and 100 μL DMSO added to dissolve formazan precipitate. Well contents were gently mixed by shaking and optical densities read same as above at 595 nm. Percentage inhibition was calculated using the formula below:

$$ \%\mathrm{Inhibition}=\frac{\mathrm{OD}\ \mathrm{of}\ \mathrm{Negative}\ \mathrm{control}-\mathrm{OD}\ \mathrm{of}\ \mathrm{extract}}{\mathrm{OD}\ \mathrm{of}\ \mathrm{Negative}\ \mathrm{control}}\ \mathrm{X}\ 100 $$

CC_50_ of the cytotoxic extract was determined by plotting a graph, using Graph pad prism, of % inhibition against log of concentration of extracts.

### Acute oral toxicity test for TP2 extract

This was done following guidelines of the Organization for Economic Cooperation and Development version 423 [22]. This was done for the bactericidal extract TP2 as described [23]. Five adult female BALB/c mice (nine weeks old) were selected in the animal house of the study laboratory and the experiment conducted in the adjacent test room. One animal was weighed, fasted overnight (no food; water only), and administered a dose of 2000 mg/kg body weight by oral gavage from a freshly prepared solution of TP2 in 7% Tween 80. The animal was fasted for a further 2 h, then provided food and water. The treated animal was observed for any obvious acute signs of toxicity hourly during the day while the other four were fasted overnight. Following the survival of the treated animal after 24 h, the other four fasted animals were weighed and treated as above and all animals observed for gross changes such as loss of appetite, piloerection, lacrimation, tremors, convulsions, salivation, diarrhoea, mortality and other signs of overt toxicity for 14 days. The animals were then sacrificed by cervical dislocation.

### Data analysis

Zone diameters recorded in the susceptibility test and antibacterial screening by disc diffusion were interpreted based on CSLI reference data for the corresponding antibiotics [20]. The zone diameters of the extracts were compared with corresponding values for the Gentamycin positive control at a significance of *P* < 0.05 using the T-test (data with normal distribution determined with the Kolmogorov-Smirnov-Test) or Welch test (no normal distribution of data). In the microdilution assay, the % growth of bacteria calculated from optical densities were plotted against concentration of extract, then MIC and MBC interpolated at the lowest of the concentrations at which bacterial growth was near constant and no longer decreasing. Data from cytotoxicity assay was analysed using Graph pad prism version 6.0, the concentration of extract that produced 50% cytotoxic effect (CC_50_) on Monkey kidney epithelial cells obtained.

## Results

### Information on remedies

Of the ten remedies purchased, two (200 Disease cure and German powder), which could not be identified were excluded from the study. Of the eight remedies investigated, six were powder and two in liquid form. Information on the remedies obtained from the sellers and the corresponding taxonomic information are shown on Table 1. The sources revealed that some remedies originate far from the local markets where sold, within Cameroon and abroad from neighbouring Nigeria and further from the republic of Niger, West Africa. Following solvent extraction of the powders high yields were obtained for most of the extracts ranging from 31 to 81%. The taxonomic information of the plant species contained in four remedies were established as described under the methods section, based on their corresponding names (Madachi, Bagaruwa, Gewaya tsamiya and Gesa) in the Hausa vernacular of the source areas and specimens provided.

**Table 1:** Traditional anti-infective remedies purchased in Buea, Cameroon

Product Name	Source/ Use	Component(s)		Remedy Extract	
		Family, Species (Voucher number)/ or Reference	Local name (part used)	Code Yield (%)	Phytochemical classes
Kiankia	SW Cameroon/ typhoid fever, malaria	Annonaceae: *Annickia chlorantha* (Oliv.) Setten & Maas (SCA 1043)	Quinine stick (stem bark)	TP1 50.6	Alkaloids (3) Triterpenoids (2) Flavonoids (1) Tannins (1)
		Apocynaceae *Alstonia boonei* De Wild (SCA 5972)	Milk stick (stem bark),		
Madachi ^a^	Nigeria and Niger/ gastritis, stomach irritation	Meliaceae: *Khaya senegalensis* (Desv.) A. Juss) SCA 6785 [40, 41]	Mahogany (stem bark)	TP2 31.0	Alkaloids (3) Triterpenoids (3) Flavonoids (2) Tannins (3)
Madigest 2	NW Cameroon/ gut infection, gastritis	Liliaceae: *Allium sativum* (SCA 1143)	Garlic (fruit),	TP3 62.4	Alkaloids (3) Flavonoids (2) Steroids (3) Tannins (2)
		Zingiberaceae: *Zingiber officinale* (SCA2926)	Ginger (rhizome)		
		Asphodelaceae: *Aloe vera* (SCA 713)	*Aloe vera* (leaves)		
Desert war 2	NW Cameroon/ microbial iinfections plus gonorrhoea, syphilis, urinary tract pains	Myrtaceae: *Eucalyptus globulus* Libille (SCA 7840)	Eucalyptus (leaves)	TP4 36.3	Alkaloids (3) Flavonoids (2) Steroids (3) Tannins (1)
		Liliaceae: *A. sativum* (SCA 1143)	Garlic (fruit)		
		Araliaceae: *Vernonia guineensis* (SCA12431)	Cameroonian ginseng (rhizome)		
		Asphodelaceae: *Aloe vera* (SCA 713)	*Aloe vera* (leaves)		
Bagaruwa^a^	North and NW Cameroon/ Cough	Fabaceae: *Acacia nilotica* (L.) Willd. ex Delile (SCA 2973) [40, 42]	^a^ (stem bark)	TL5	Triterpenoids (2) Flavonoids (3) Tannins (3)
Gewaya tsamiya ^a^	Niger / typhoid fever, malaria, yellow fever	Fabaceae: *Cassia nigricans* Vahl [40, 41] (N^O^ 5970/HNC)	^a^ (leaves)	TP6 56.8	Alkaloids (3) Triterpenoids (3) Flavonoids (3) Steroids (3) Tannins (3)
Fever medicine	SW Cameroon/ malaria/typhoid fever	Labiatae: *Ocimum gratissimum* (SCA 4764)	Masepu (leaves)	TL7	Triterpenoids (2) Flavonoids (2) Tannins (2)
		Asteraceae: *Bidens pilosa* (SCA 6352)	Black jack (leaves)		
		Caricaceae: *Carica papaya* (SCA 1756)	Paw paw (leaves)		
		Rutaceae: Citrus *aurantifolia* (SCA11102)	Limes (fruit)		
Gesa^a^	North and NW Cameroon/ diarrhoea	Combretaceae: *Combretum micranthum* (SCA 2976) [40, 42]	^a^ (leaves)	TP8 81.5	Alkaloids (2) Triterpenoids (3) Flavonoids (3) Tannins (3)

SW Southwest, NW Northwest, TP Crude extract prepared from powder remedy, TL Liquid remedy used neat without further extraction. ^a^Local name in Hausa vernacular [40, 41], Bagaruwa has as synonym Gabaruwa in some reports [41]. Relative amount:- 1 = low; 2 = moderate; 3 = high

### Microbial content of remedies

All eight remedies were contaminated with both Gram- positive and negative bacilli and cocci. The bacterial load ranged from 1.8 × 10^3^ to 2.1 × 10^5^ CFUs/mg or CFUs/mL. None had a microbial count within the acceptable range of microbial contamination of 10^5^ CFU/g (10^2^ CFU/mg) for aerobic bacteria as stipulated in the WHO guidelines on the quality of herbal materials [24]. TP3 (Madigest 2) and TP4 (Desert war 2) had the highest microbial counts of 1.8 × 10^5^ CFU/mg and 2.1 × 10^5^ CFU/mg respectively while TP7 (Fever medicine) had the lowest microbial count of 1.8 × 10^3^ CFU/mg.

### Antibiotics susceptibility of bacteria

Based on reference data (diameter of inhibition zones) for antibiotic susceptibility of the Clinical and Laboratory Standards Institute [20], both the control and clinical isolates were sensitive to the aminoglycosides (Amikacin, Gentamicin), fluoroquinolones (Ciprofloxacin, Norfloxacin), Nitrofurantoin and Chloramphenicol. Resistance was observed for the carbapenem (Imipenem), cephems (Cefotaxime, Ceftraixone, Cefuroxime), folate pathway inhibitor (Trimethoprim) and Tetracycline. Each bacterial strain was resistant to at least one antibiotic. Multidrug resistance was confirmed in nine of the ten clinical isolates (resistant to at least one antibiotic of three chemical classes) [2], presented on Table 2. *Proteus vulgaris, Providencia rettgeri* and *Salmonella typhi* were the most resistant to 5, 6 and 5 classes of antibiotics respectively. The resistance of the clinical isolate of *S. aureus* to methicillin was confirmed.

**Table 2:** Susceptibility of bacterial strains to standard antibiotics

Bacteria Strains	Diameter of zone of inhibition (mm)												Resistant classes of antibiotics (n)
	AK	GM	CI	NX	CL	NI	IP	TX	CT	XM	TR	TE	
*Citrobacter freundii*	27	24	30	32	23	25	–	–	–	–	–	15	3
*Citrobacter youngae*	30	28	31	31	27	25	–	–	–	–	11	–	3
Citrobacter sp.	19	16	20	26	21	22	–	17r	–	–	30	16	2
*Enterobacter cloacae*	23	17	30	30	24	16	15r	15r	–	–	27	–	3
*Escherichia coli*	25	18	–	10r	22	30	25	–	–	–	–	–	4
*MRSA*	24	22	35	29	33	24	ND	10r	–	ND	15	15	3^b^
*Proteus mirabilis*	20	20	37	36	–	14r	–	23	–	–	22	–	4
*Proteus vulgaris*	20	16	28	30	30	14r	15r	25	–	–	–	–	5
*Providencia rettgeri*	20	19	25	27	–	–	–	–	–	–	–	–	6
*Salmonella typhi*	19	20	25	25	–	15	–	–	–	–	–	–	5
Salmonella sp.	20	15	36	37	23	18	18r	–	–	–	–	17	3
*E. coli* ^a^	ND	20	26	30	29	22	8r	8 r	ND	ND	8 r	8 r	4
*P. aeruginosa* ^a^	ND	24	30	24	18	20	–	8	ND	ND	–	20	1
*S. enterica* ^a^a	ND	22	20	28	30	21	–	20	ND	ND	22	19	1
*S. enterica*^a^b	ND	19	29	27	25	9r	9 r	–	ND	ND	11r	19	3
*S. aureus* ^a^	ND	21	22	19	15r	12r	7r	8r	ND	ND	7 r	19	5
*S. epidermidis* ^a^	ND	22	ND	29	28	7r	0r	10r	ND	ND	29	28	3

Antibiotic classes: Aminoglycosides (AK Amikacin, GM Gentamicin), Carbapenems (IP Imipenem), Cephems (CT Cefotaxime, TX Ceftriaxone, XM Cefuroxime), Fluoroquinolones (CI Ciprofloxacin, NX Norfloxacin), Folate pathway inhibitors (TR Trimethoprim), Nitrofurans (NI Ntrofurantoin), Phenicols (CL Chloramphenicol), Tetracyclines (TE Tetracycline)

^a^ Control strains (BEI Resources and ATCC, USA); *S. enterica* strains a = (NR-515), b = (NR-4311). MRSA: methicillin resistant *Staphylococcus aureus*; ^b^ resistant to methicillin, confirmed by a 0 mm zone not shown on the table. r and - (zone diameter = 0 mm), both indicate resistant strain. n: Number of antibiotic classes to which bacterial strain is resistant based on the reference data [20]; *n* ≥ 3, strain is considered multidrug resistant [2].

### Antibacterial activity of remedies

**Fig. 1:**
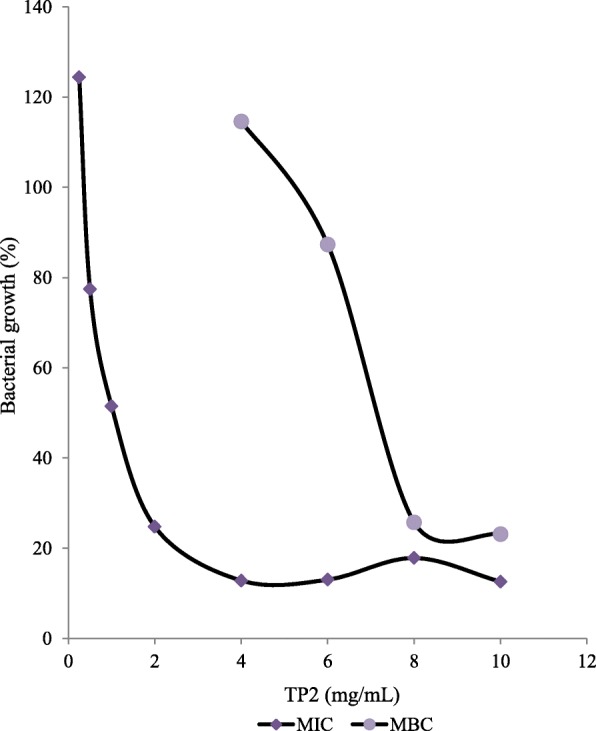
Effect of TP2 extract on growth of methicillin resistant *Staphylococcus aureus*. Legend: TP2 is methanol extract of Madachi traditional remedy prepared from stem bark *Khaya senegalensis*. MIC and MBC are minimum inhibitory and minimum bactericidal concentrations respectively

Extracts of four remedies (TP 1, 2, 4, 6a, 6b) were active against all the clinical isolates. Extracts of three remedies (TP 1, 2, 4, 6a, 6b) were active against at least four of the control strains. All extracts showed activity against *S. enterica* (NR-515). The liquid remedies (TL 5 and 7) tested undiluted were less active; TL7 showed relatively low activity against only two control strains with no activity against the clinical isolates (Table 3). The zones of inhibition for the extracts ranged from 8 to 27 mm against 17 to 24 mm for Gentamycin positive control. Two extracts (TP1 and 4) produced zones ≥20 mm against multidrug resistant clinical isolates (*Citrobacter freundii* and *Escherichia coli*). The highest zone against clinical isolates was 24 mm produced by TP1 against *E. coli*; and the highest zone against control strains was 27 mm produced by TP4 against *Staphylococcus aureus* (NR-46003). However, when zone diameters were compared using statistical tests, Gentamycin was more active than each of the four most active extracts (*P* < 0.0001–0.0014). Four of the eight remedies (TP 1, 2, 4, 6) recorded MIC values from 1 to 4 mg/mL. TP1 had the lowest MIC of 1 mg/mL against *S. typhi* clinical isolate shown on Table 4. Of these 4 extracts, only TP2 showed MBC at 8 mg/mL against MRSA. The MBC:MIC ratio gave a value of 2 which is less than 4 indicating bactericidal effect of TP2 against this strain (Table 4) [25]. Figure 1 shows inhibition of growth at higher concentrations of TP2 with higher growth at the lower concentrations giving the MIC and MBC values of 4 and 8 mg/mL respectively.

**Table 3:** Antibiogram of methanol extracts of anti-infective traditional remedies sold in Buea, Cameroon

Bacteria	Diameter of zone of inhibition (mm)									
	TP1	TP2	TP3	TP4	TP5	TP6a	TP6b	TP7	TP8	GM
*Citrobacter freundii*	12	13	10	20	–	11	11	–	10	23
*Citrobacter youngae*	11	11	11	13	10	10	11	–	–	23
*Enterobacter cloacae*	19	12	14	11	–	17	16	–	10	24
*Escherichia coli*	24	15	12	13	10	11	12	–	15	21
MRSA	15	–	10	11	9	11	12	–	11	10^a^,37^b^
*Proteus mirabilis*	15	17	14	13	8	15	15	–	9	24
*Proteus vulgaris*	22	11	10	13	9	11	11	–	9	17
*Providencia rettgeri*	15	13	13	12	–	12	10	–	9	20
*Salmonella typhi*	13	13	–	17	–	10	10	–	–	17^c^
^f^ *E. coli*	–	14	–	11	–	9	11	9	–	18
^f^ *P. aeruginosa*	15	15	11	16	9	11	11	–	10	20
^f^ *S. aureus*	13	12	13	27	–	16	11	–	13	24
^f^ *S. enterica* ^d^	13	11	12	15	13	9	10	12	15	20
^f^ *S. enterica* ^e^	–	–	–	–	–	–	–	–	–	8^a^

TP Extract of powder remedy, TL Liquid remedy tested undiluted. Positive controls: GM Gentamicin, a: Norfloxacin; b: Trimethoprim; c: Chloramphenicol. Nd Not Done. -: No inhibition. TP6a: methanol extract residue (deposited below oil layer in extract container), TP6b: oil layer above solid residue of TP6 crude extract. MRSA: methicillin resistant *Staphylococcus aureus*. ^f^Control strains; *S. enterica* strains d = (NR-515), e = (NR-4311)

**Table 4:** MICs, MBC and CC_50_ of crude extracts of traditional medicine preparations

Product Name	Extract code	Activity (Organism)		CC_50_ (μg/mL)
		MIC (mg/mL)	MBC (mg/mL)	
Kiankia	TP1	1 (ST)	–	652.3
Madachi ^a^	TP2	4 (MRSA)	8 (MRSA)	689.0
Desert war 2	TP4	4 (MRSA)	–	218.3
Bagaruwa^a^	TL5	–	–	11.4
Gewaya tsamiya ^a^	TP6b	4 (PM)	–	435.2
Fever medicine	TL7	–	–	149.2
Gesa^a^	TP8	–	–	502.1

MIC Minimum inhibitory concentration, MBC Minimum bactericidal concentration, CC_50_ Cytotoxic concentration for 50% of monkey kidney epithelial cells (LLC-MK2 from ATCC, Virginia, USA).. ST *Salmonella. typhi*, MRSA Methicillin resistant *Staphylococcus aureus*, PM *Proteus mirabilis*.TP6b: oil layer above solid residue of TP6 crude extract. -: no MIC, no MBC recorded in the test concentration range. ^a^Local name in Hausa vernacular

### Phytochemical profile of extracts

The crude extracts and undiluted liquid remedies were found to contain alkaloids, flavonoids, steroids, tannins and triterpenoids from the phytochemical tests. The four most active extracts (TP 1, 2, 4, 6a, 6b) had relatively high amounts of alkaloids and flavonoids (Table 1).

### Toxicity of remedies

The cytotoxicity assay on monkey kidney cells, LLCMK2 Original (ATCC® CCL7™), showed that 2 extracts (TP 3 and 6a) were non-cytotoxic at a high dose of 1000 μg/mL since their percentage inhibition of formazan formation was less than 50%. Six extracts (TP 1, 2, 4, 6b, 8 and TL7) had CC_50_ values greater than the cut-off point for lack of cytotoxicity (CC_50_ > 30 μg/mL), [26]. One extract (TL5) had CC_50_ value lower than 30 μg/mL indicating mild cytotoxicity (Table 4). Only piloerection was observed in the first two days in the oral toxicity test for TP2 with no mortality recorded. The body weight of all the animals increased; average body weights at the start and after day 14 were 26.6 and 29.4 g respectively with an average increase of 2.8 g.

## Discussion

Traditional medicine remedies are commonly sold in local markets in Cameroon and some may serve as alternative therapy for infections caused by multidrug resistant bacteria or source of new efficacious antibacterial compounds. Three main findings were recorded in this study of eight anti-infective traditional remedies sold in Buea, Cameroon. Firstly, only four (50%), showed activity against resistant bacteria based on minimum inhibitory concentration (MIC) recorded on at least one bacterial strain. Secondly and contrary to the above, a promising finding was made in that six of seven remedies tested for cytotoxicity were not cytotoxic, suggesting that these remedies may not cause serious toxicity when used for therapeutic purposes. Thirdly, all eight remedies were found to be contaminated by bacteria above acceptable limits.

Nine of the ten bacteria isolates tested for antibiotic susceptibility were multidrug resistant. This finding confirmed that of the medical laboratory of the Buea Regional Hospital where they were isolated from clinical specimens and first tested for antibiotic susceptibility (Table 2). These data also confirm findings of previous studies in the study area which reported high levels of resistance in *Citrobacter freundii*, *Enterobacter cloacae*, *P. aeruginosa* and *Proteus vulgaris*, whereby more than 50% of isolates from clinical specimens were resistant to antibiotics [27]. The information provided by sellers of the remedies indicates that all the remedies are used to treat bacterial infections among others (Table 1), thereby justifying the screening of these remedies for antibacterial activity against multidrug resistant strains.

Overall, the four most active extracts (TP 1, 2, 4, 6a, 6b) showed bacteriostatic activity, and also showed the highest activity against an individual bacteria strain in both the disc diffusion test and the MIC assay. These extracts produced the highest zones ranging from 16 mm for TP6b to 27 mm for TP4, in the disc diffusion test. However, when all the zones for each extract were considered (Table 3), they were significantly (*P* < 0.0001–0.0014) lower than those of the Gentamycin positive control suggesting the extract are less active than Gentamycin. These four extracts also produced the lowest MICs (1 mg/mL for TP1 and 4 mg/mL for the others) showing consistency in their activity in the disc and microdilution assays (Tables 3 and 4). Furthermore, one of them (TP2) also recorded a MBC value (8 mg/mL) with the MIC:MBC ratio equal to 2 (being less than 4) indicating bactericidal activity [25], (Fig. 1). The findings of this study show that the four most active remedies are potential alternative or complementary antibacterials for treatment of infections of resistant bacteria. However, activity was observed for only four of eight remedies suggesting some of the remedies may not be sufficiently efficacious in clinical infections. They should be further investigated using in vivo experiments to evaluate the efficacy of the remedies.

The cytotoxicity test showed that six of the remedies have a low risk of toxicity as seen from their CC_50_ values of 149 to 689 μg/mL (Table 4), which are much higher than the cut-off point for lack of cytotoxicity (CC_50_ > 30 μg/mL), [26]. When the United States National Cancer Institute plant screening program criterion for cytotoxic (CC_50_ is ≤20 μg/mL) is considered, only one extract TL5 (CC_50_ = 11.4 μg/mL) is cytotoxic [28]. This finding supports the general view that traditional remedies are safe though this must necessarily be confirmed by experimental data, given the increasing evidence of their toxicity [29]. One of the most active and bactericidal extract TP2, recorded the highest CC_50_ (689 μg/mL, Table 4) indicating a very high safety margin. This is further supported by findings in the oral toxicity test where no mortality occurred after 14 days at 2000 mg/kg body weight, with only piloerection observed in the first two days. Piloerection (involuntary erection or bristling of hairs) is likely due to sympathetic stimulation involving body heat or temperature regulation mechanisms or due to serotonin release [30].

Whereas the toxicity data for almost all the remedies are favourable, the microbial contamination is the reverse being higher (1.8 × 10^3^ to 2.1 × 10^5^ CFUs/mg or mL) than the acceptable limit of 10^5^ CFU/g (10^2^ CFU/mg) in herbal materials [24]. Hence the remedies ironically have a potential risk of infection to the consumers. This risk may be averted through neutralization of the microbes by acidic gastric secretion following oral administration which is the principal route for these remedies. A study found high Bacillus spp. contamination (1.2 × 10^3^ to 6 × 10^5^ CFUs/g or mL) in ten herbal remedies manufactured by a business entity and sold widely [31]. Similar findings in another study were attributed to poor preparation and handling of these remedies [10]. The distant source of some of the remedies implies a long chain of custody which may aggravate the contamination. At the user end, some of the products are displayed in open containers and even when closed, are sold by rationing from the same stock hence favouring further contamination by the handlers and the surrounding. The preparation and handling of these products therefore needs significant improvement, standardisation and quality control at all stages to commercialization. The distant source also made establishment of the components of two remedies (excluded from the study) daunting and untraceable as the sellers acquire their stock from middlemen who also do not have this information.

Interestingly, the yields of the powder extracts were quite high reaching about 80% (Table 1), probably due to thorough drying. Phytochemical analysis revealed the most active remedies were rich in alkaloids with also moderate to high amounts of flavonoids and triterpenoids. Antimicrobial activity of extracts of the plants contained in the most active remedies has been reported; phytochemicals of classes mentioned above among others have been detected in them and the structures of some of their bioactive secondary metabolites reported. These include *A. boonei* [32]; *K. senegalensis* [33]; *A. vera* [34] and *C. nigricans* [35]; this study therefore further confirms the reported activities. The activity of the traditional remedy (Madachi) is likely accounted for by one or more of the compounds detected in the TP2 extract as earlier reported for *Khaya senegalensis* [33]. The possible mechanisms of action of the active extracts will be investigated later. However, some studies have revealed a number of mechanisms of action of extracts on bacteria. These include decrease in bacterial cytoplasmic pH and disruption of cell wall [36], generation of reactive-oxygen species and membrane damage [37]. Some extracts cause potentiation of the action of antibiotics resulting in a synergistic inhibition of bacterial cell wall synthesis hence afford a possibility of combination therapy with antibiotics in clinical use [38].

## Conclusion

Only four of the eight remedies tested showed activity against multidrug resistant bacteria suggesting some of these remedies may not be effective against bacterial infections. However, most of the remedies have a low risk of toxicity in spite of their high microbial content. The production and handling methods should be improved to prevent microbial contamination; and the remedies subjected to stringent quality control before commercialization to ensure biosecurity. Considering their activity against multidrug resistant bacteria and the low risk of toxicity, further studies should be done including in vivo experiments to assess their efficacy.

## Data Availability

The datasets used and or analysed in this study are available from the corresponding author on reasonable request.

## Abbreviations

CC_50_: Concentration which kills 50% of cells
CCM: Complete culture medium containing serum
CFU: Colony forming units
DMSO: Dimethyl sulfoxide
ICM: Incomplete culture medium lacking serum
MBC: Minimum bactericidal concentration
MIC: Minimum inhibitory concentration
MRSA: Methicillin-resistant *Staphylococcus aureus*
MTT: A tetrazolium bromide chromogen
OD: Optical density
TL: Code for liquid remedy tested pure
TP: Extract code for powder remedy
WHO: World Health Organization

## References

[1] World Health Organization. The top 10 causes of death https://www.who.int/news-room/fact-sheets/detail/the-top-10-causes-of-death. Accessed 5 Feb 2019.

[2] Magiorakos AP, Srinivasan A, Carey RB, Carmeli Y, Falagas ME, Giske CG (2012). Multidrug-resistant, extensively drug-resistant and pandrug-resistant bacteria: an international expert proposal for interim standard definitions for acquired resistance. Clin Microbiol Infect.

[3] Ventola CL (2015). The antibiotics resistance crisis. Part 1: causes and threats. P &T.

[4] Bassetti M, Maria M, Chiara T, Augusta A (2013). New antibiotics for bad bug: Where are we?. Ann Clin Microbiol Antimicrob.

[5] Piddock LJV (2015). Teixobactin, the first of a new class of antibiotics discovered by iChip technology?. J Antimicrob Chemother.

[6] Essack SY, Desta AT, Abotsi RE, Agoba EE (2016). Antimicrobial resistance in the WHO African region: current status and roadmap for action. J Public Health.

[7] World Health Organization. WHO Traditional Medicine Strategy 2014–2023. http://www.who.int/medicines/publications/traditional/trm_strategy14_23/en/. Accessed 4 June 2017. (2013)

[8] Fokunang CN, Ndikum V, Tabi OY, Jiofack RB, Ngameni B, Guedje NM, Kamsu-Kom (2011). Traditional medicine: past, present and future research and development prospects and integration in the national health system of Cameroon. Afr J Tradit Complement Altern Med.

[9] Tambekar DH, Dahikar SB (2010). Antibacterial potential of some herbal preparation: an Alternative medicine in treatment of enteric bacterial infection. Int J Pharm Pharm Sci.

[10] Oyetayo VO (2008). Microbial load and antimicrobial property of two Nigerian herbal remedies. Afr J Tradit Complement Altern Med.

[11] Osei-Djarbeng SN, Nyarko S, Osei-Asante S, Owusu-Dapaah G (2016). Absence of *in-vitro a*ntimicrobial activity of some antimicrobial herbal preparations in the Ghanaian market. J Pharmacogn Phytochem.

[12] Abdualmjid RJ, Sergi C (2013). Hepatotoxic Botanicals - An evidence-based systematic review. J Pharm Pharmaceut Sci.

[13] Chusri S, Sinvaraphan N, Chaipak P, Luxsananuwong A, Voravuthikunchai SP (2014). Evaluation of antibacterial activity, phytochemical constituents, and cytotoxicity effects of Thai household ancient remedies. J Altern Complement Med.

[14] Kim Hong, Jang Eungyeong, Kim So-Young, Choi Ji-Yoon, Lee Na-Rae, Kim Dae-Sung, Lee Kyung-Tae, Inn Kyung-Soo, Kim Bum-Joon, Lee Jang-Hoon (2018). Preclinical Evaluation of In Vitro and In Vivo Antiviral Activities of KCT-01, a New Herbal Formula against Hepatitis B Virus. Evidence-Based Complementary and Alternative Medicine.

[15] Wilmot D, Ameyaw EO, Amoako-Sakyi A, Boampong JN, Quashie NB (2017). *In vivo* efficacy of top five surveyed Ghanaian herbal anti-malarial products. Malar J.

[16] Ferrazzano Gianmaria Fabrizio, Cantile Tiziana, Roberto Lia, Ingenito Aniello, Catania Maria Rosaria, Roscetto Emanuela, Palumbo Giuseppe, Zarrelli Armando, Pollio Antonino (2015). Determination of theIn VitroandIn VivoAntimicrobial Activity on Salivary Streptococci and Lactobacilli and Chemical Characterisation of the Phenolic Content of aPlantago lanceolataInfusion. BioMed Research International.

[17] Cheesbrough M (2006). District Laboratory Practice in Tropical Countries Part II.

[18] Mbah JA, Ngemenya MN, Ashime LA, Babiaka SB, Nubed LN, Nyongbela KD (2012). Bioassay-guided discovery of antibacterial agents: *in vitro s*creening of *Peperomia volcanic*, *Peperomia fernandopoioana* and *Scleria striatinux*. Ann Clin Microbiol Antimicrob.

[19] Ram J, Moteriya P, Chanda S (2015). Phytochemical screening and reported biological activities of some medicinal plants of Gujarat region. J Pharmacogn Phytochem.

[20] Clinical and Laboratory Standards Institute (2012). Performance standards for antimicrobial susceptibility testing; twenty second informational supplement M100 – S22.

[21] Nondo RSO, Moshi MJ, Paul Erasto P, Zofou D, Njouendou AJ, Wanji S (2015). Evaluation of the cytotoxic activity of extracts from medicinal plants used for the treatment of malaria in Kagera and Lindi regions,Tanzania. J Appl Pharm Sci.

[22] Organization for Economic Cooperation and Development. OECD Guideline for testing of chemicals. Version 423 Adopted: 17 December 2001. http://www.oecd.org/chemicalsafety/testing/oecdguidelinesforthetestingofchemicals.htm Accessed 4 June 2017

[23] Fentahun S, Makonnen E, Awas T, Giday M (2017). *In vivo* antimalarial activity of crude extracts and solvent fractions of leaves of *Strychnos mitis* in *Plasmodium berghei* infected mice. BMC Complement Altern Med.

[24] World Health Organization (2007). WHO guidelines for assessing quality of herbal medicines with reference to contaminants and residues.

[25] Haas W, Pillar CM, Hesje CK, Sanfilippo CM, Morris TW (2010). Bactericidal activity of besifloxacin against staphylococci, *Streptococcus pneumoniae* and *Haemophilus influenza*. J Antimicrob Chemother.

[26] Malebo HM, Tanja W, Caletal M, Omolo MO, Hassanali A (2009). Antiplasmodial, antitrypanosomal, antileshmanial and cytotoxicity activity of selected Tanzanian medicinal plants. Tanzan J Health Res.

[27] Akoachere JFTK, Yvonne S, Akum NH, Esemu NS (2012). Etiologic profile and antimicrobial susceptibility of community-acquired urinary tract infection in two Cameroonian towns. BMC Res Notes.

[28] Malek SNA, Phang CW, Ibrahim H, Norhanom AW, Sim KS (2011). Phytochemical and cytotoxic investigations of *Alpinia mutica* rhizomes. Molecules.

[29] Ifeoma O, Oluwakanyinsola S (2013). Screening of herbal medicines for potential toxicities.

[30] Matsumotoa RR, Seminerioa MJ, Turnerb RC, Robsona MJ, Nguyena L, Millerf DB (2014). Methamphetamine-induced toxicity: an updated review on issues related to hyperthermia. Pharmacol Ther.

[31] Shah B, Pokhrel N (2012). Microbial quality and antibacterial activity of herbal medicines. NJST.

[32] Adotey John Prosper Kwaku, Adukpo Genevieve Etornam, Opoku Boahen Yaw, Armah Frederick Ato (2012). A Review of the Ethnobotany and Pharmacological Importance of Alstonia boonei De Wild (Apocynaceae). ISRN Pharmacology.

[33] Paritala V, Chiruvella KK, Chakradhar Thamminenic C, Ghantad RG, Mohammed A (2015). Phytochemicals and antimicrobial potentials of mahogany family. Rev Bras Farmacogno.

[34] Nejatzadeh-Barandozi F (2013). Antibacterial activities and antioxidant capacity of *Aloe vera*. Org Med Chem Lett.

[35] Ayo RG (2010). Phytochemical constituents and bioactivities of the extracts of *Cassia nigricans V*ahl: A review. J Med Plants Res.

[36] Gonelimali FD, Lin J, Miao W, Xuan J, Charles F, Chen M, et al. Antimicrobial properties and mechanism of action of some plant extracts against food pathogens and spoilage microorganisms. Front Microbiol. 2018;(9):1639. 10.3389/fmicb.2018.01639. 10.3389/fmicb.2018.01639 PMC6066648 30087662

[37] Tang Q-L, Kang A-R, Lu C-X (2016). Phytochemical Analysis, Antibacterial Activity and Mode of Action of the Methanolic Extract of *Scutellaria barbata* Against Various Clinically Important Bacterial Pathogens. Int J Pharmacol.

[38] Voukeng IK, Kuete V, Dzoyem JP, Fankam AG, Noumedem JAK, Kuiate JR (2012). Antibacterial and antibiotic-potentiation activities of the methanol extract of some Cameroonian spices against Gram-negative multi-drug resistant phenotypes. BMC Res Notes.

[39] Directives. Directive 2010/63/EU of the European Parliament and of the Council of 22 September 2010 on the protection of animals used for scientific purposes. *OJ* 2010, L 276/33. https://en.wikipedia.org/wiki/EU_Directive_2010/63/EU. Accessed 4 June 2017.

[40] De Fabregues BP (1977). Lexique des noms vernaculaires de plantes du Niger. 2e edition provisoire. 1977.

[41] Prelude medicinal plants database. http://www.africamuseum.be/collections/external/prelude/view_plant?pi=02630 Accessed 5 July 2017.

[42] Tukur R, Adamu GK, Rabi’u M AI (2013). Indigenous trees inventory and their multipurpose uses in Dutsin-ma area Katsina state. Eur Sci J.

